# Residual Life Prediction of XLPE Distribution Cables Based on Time-Temperature Superposition Principle by Non-Destructive BIS Measuring on Site

**DOI:** 10.3390/polym14245478

**Published:** 2022-12-14

**Authors:** Bingliang Shan, Chengqian Du, Junhua Cheng, Wei Wang, Chengrong Li

**Affiliations:** State Key Laboratory of Alternate Electrical Power System with Renewable Energy Sources, North China Electric Power University, Beijing 102206, China

**Keywords:** XLPE cable, aging trend, residual life, time-temperature superposition principle

## Abstract

Crosslinked polyethylene (XLPE) distribution cables are prone to segmented thermal aging after long-term operation owing to the large spatial spans and complex operating environments, and accurate residual life prediction of each aging cable segment could provide a theoretical basis and reference for performance monitoring, maintenance and the replacement of cables. Existing studies mainly focus on the residual life prediction methods for uniform aging cables, which are not suitable for segmented-aging cables. In this paper, a residual life prediction method for segmented-aging XLPE distribution cables based on the time-temperature superposition principle (TTSP) by non-destructive BIS measuring on site was proposed. Firstly, the applicability of the TTSP in the transformation of the changing process of elongation at break (EAB) of XLPE at different thermal aging temperatures was verified based on the Arrhenius equation. Secondly, to better simulate the thermal aging process under working conditions, XLPE cables were subjected to accelerated external stress aging at 140 °C for different aging times, and the corresponding changing process of EAB along with aging time was further measured. The relationship between the EAB of XLPE cables and aging time was well fitted by an equation, which could be used as a reference curve to predict the thermal aging trends and residual life of service-aged XLPE cables. After that, a calculation method for the transformation of the changing process of EAB of XLPE at different thermal aging temperatures was proposed, in which the corresponding multiplicative shift factor could be obtained based on the TTSP instead of the Arrhenius equation extrapolation. Moreover, the availability of the above calculation method was further proved by accelerated thermal aging experiments at 154 °C; the results show that the prediction error for the cable’s EAB is no more than 3.15% and the prediction error for residual life is within 10% in this case. Finally, the realization of non-destructive residual life prediction combined with BIS measuring on site was explained briefly.

## 1. Introduction

Cross-linked polyethylene (XLPE) cables possess excellent electrical and mechanical properties, which have been widely used in distribution network construction in China [1]. Some distribution cables put into operation earlier are gradually approaching their design life, and the aging problem of cable insulation caused by long-term operation should not be ignored since it threatens the operation safety of the distribution network [2]. Owing to the large space spans and complex operating environments of the actual XLPE distribution cables, the segmented thermal aging phenomenon occurs easily, causing the non-uniform distribution of the aging status and residual life along the cables [3]. In order to optimize urban construction structures and improve urban appearances, the majority of distribution cables are chosen to be laid underground; in this situation, it is not conducive to evaluate the cable aging status and to predict the residual life by destructive methods. Thus, it is of great significance to realize the differential residual life prediction of segmented-aging distribution cables in service by non-destructive methods, based on which the maintenance strategy and timely retirement plans of distribution cables could be formulated and implemented accordingly.

Thermal aging can cause some changes in the physical and chemical properties of XLPE cable insulation [4]. Following this, some methods for characterizing the status of thermal aging cables have been developed in recent years, such as dielectric loss measuring [5], oxidation induction time testing [6], infrared absorption spectra [7], EAB [8] and so on. Among them, EAB has attracted more and more attention since it can reflect the aging condition of cable insulation more effectively and sensitively. Crine et al. [9] studied the effect of thermal aging of XLPE cables on EAB of the insulation layer and they found that the thermal aging of cables can result in the decrease of EAB of the insulation layer. Other work [10] carried out accelerated aging experiments on slice samples of XLPE cable insulation at different thermal aging temperatures; the results showed that the speed of cable aging varies with the aging temperature. That is, the elevation of the aging temperature set accelerates the decreasing speed of EAB of slice samples. The standard IEC: 60502-2 (2014) [11] proposed that the EAB could be used as an evaluation index for the performance deterioration and residual life of XLPE cables.

In fact, the question of how to accurately predict the aging trend of the cable insulation is the core of life prediction. It has been proved that the Arrhenius equation can be used to describe the degradation process of some certain materials quantitatively and realize the transformation of life characteristics under different stress levels [12]. Kenneth T. Gillen et al. carried out oven aging studies on polyethylene cable jacket materials; the mechanical property results of some of the materials showed reasonable Arrhenius behavior [13]. The degradation curves (plotted versus log of the aging time) based on EAB at differing aging temperatures possess similar shapes, and could be converted into each other by using empirical shift factors. Meng Xiaokai utilized tensile elongation as the degradation parameter to analyze the aging process of the ethylene-propylene rubber (EPR) insulation used in shipboard power cables at different aging temperatures; an extrapolation equation for the lifetime prediction of the EPR insulation material according to the Arrhenius equation was then established [14]. The above research results provide guidance for predicting the thermal aging trend of XLPE distribution cables in operation. Nevertheless, the Arrhenius equation is rarely used to convert the changing process of EAB of XLPE cable insulation at differing aging temperatures, the applicability of which remains unclear. Meanwhile, EAB measuring is a destructive method; it would seriously damage the integrity of cables and lead to an increase of cable failure rate, which is not advisable in engineering applications [15]. Moreover, it needs to be noted that the existing research are all aimed at residual life prediction of uniform aging cables; no work has focused on the non-destructive residual life prediction of segmented-aging XLPE cables has until now. It would hinder the rational use, maintenance, retirement and scrap of XLPE distribution cables.

In this paper, the residual life prediction of XLPE distribution cables based on the TTSP by non-destructive broadband impedance spectrum (BIS) measuring on site was explored and achieved for the first time. The applicability of the Arrhenius equation in the transformation of the changing process of EAB of XLPE materials under thermal aging conditions was verified, and the reference curve used to predict the aging trend of the service-aged cables was obtained based on the accelerated aging test at 140 °C. Then, the calculation method for the transformation of the changing process of EAB of XLPE at different thermal aging temperatures based on the TTSP was demonstrated, and its availability was further proved by accelerated thermal aging experiments at 154 °C. Finally, the realization process of non-destructive residual life prediction combined with BIS measuring on site was described in detail.

## 2. The TTSP and Applicability Analysis of the TTSP in XLPE Materials

### 2.1. Introduction of the TTSP

The TTSP is based on the observation that the short-term behavior of viscoelastic materials at higher temperatures is similar to the long-term behavior at lower reference temperatures [16]. It assumes that the effect of increasing temperature is equal to expanding the time of the creep response by a multiplicative shift factor (the temperature acceleration factor). At present, the most widely used temperature acceleration factor is one derived from the Arrhenius equation.

In fact, the Arrhenius equation is often used to describe the accelerated thermal aging process of cables quantitively, with the time when the cable is put into operation set to *t*_0_ = 0. Assuming that accelerated thermal aging occurs in a certain material at the absolute temperature *T*, and the values of the aging characteristic parameter are *M*_1_ at a given time *t*_1_ and *M*_2_ at a given time *t*_2_ (*t*_2_ > *t*_1_), the value of the variation Δ*M* from time *t*_1_ to time *t*_2_ can be calculated as follows:

(1) $$\Delta M=\left|M_{2}-M_{1}\right|=\int_{t_{1}}^{t_{2}}A_{0}e^{\frac{-E}{RT}}dt=A_{0}e^{\frac{-E}{RT}}(t_{2}-t_{1}),$$

where *E* is the activation energy (J/mol), *R* is the ideal gas constant (*R* = 8.314 J/(K·mol)) and *A*_0_ is a constant value obtained by fitting. If the material is considered to reach its end of life when the value of aging characteristic parameter decreases to $M_{end}$ and the corresponding time (lifetime) is *t*, then the following equation can be deduced according to Equation (1):

(2) $$\ln t=\ln\frac{\left|M_{end}-M_{1}\right|}{A_{0}}+\frac{E}{RT}.$$

In addition, *a* and *b* are defined in Equation (3). In view of the fact that the activation energy *E* of the material does not change when the absolute temperature is less than 500 K [17], one conclusion can easily be drawn that both *a* and *b* are fixed constants:

(3) $$\left\{ \begin{array}{l} a=\ln\frac{\left|M_{end}-M_{1}\right|}{A_{0}}\\ b=\frac{E}{R} \end{array}\right..$$

Therefore, Equation (2) can be further expressed as follows:

(4) $$\ln t=a+\frac{b}{T}.$$

It can be seen from the above equation that the natural logarithm of the cable’s lifetime *t* is linear to the inverse absolute temperature *T*. The reference temperature is *T_ref_*, and is one of the experimental temperatures set. The curve of the aging characteristic parameter versus the absolute temperature obtained at a second temperature *T*_1_ is used to find the constant multiplicative shift factor $\alpha_{T}$, such that multiplying the times associated with the temperature *T* by $\alpha_{T}$ gives the best overlap (superposition) of the data from *T* to *T_ref_*. For example, when the aging characteristic parameter reaches the same value *M*, the consuming time is *t_ref_* and *t_T_* at temperature *T_ref_* and *T*, respectively; then, the corresponding multiplicative shift factor $\alpha_{T_{1}}$ can be obtained as follows:

(5) $$\alpha_{T}=\frac{t_{ref}}{t_{T}}.$$

Combined with Equation (4), the corresponding multiplicative shift factor $\alpha_{T}$ can be further calculated by Equation (6):

(6) $$\alpha_{T}=\frac{t_{ref}}{t_{T}}=\frac{\exp(a+\frac{b}{T_{ref}})}{\exp(a+\frac{b}{T})}=\exp(\frac{E}{R}(\frac{1}{T_{ref}}-\frac{1}{T})).$$

By taking the logarithm of the left and right sides of the above equation, Equation (7) can be obtained:

(7) $$\ln\alpha_{T}=-\frac{E}{RT}+\frac{E}{RT_{ref}}.$$

It can be observed that there is a significant negative correlation between the natural logarithm of the multiplicative shift factor $\alpha_{T}$ and the inverse absolute temperature *T*. As a result, the empirically derived shift factors at different aging temperatures can be plotted on an Arrhenius plot to see if Arrhenius behavior can be observed [13].

### 2.2. Applicability Analysis of the TTSP in the Transformation of the Changing Process of EAB of XLPE at Different Temperatures

**Figure 1:**
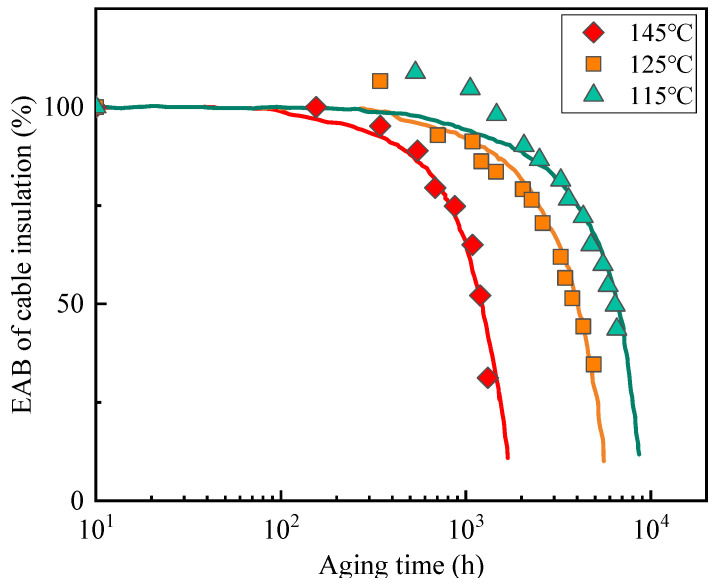
Retention rates of EAB versus aging time at the indicated temperatures for slice samples of XLPE cable insulation and the fitting curves.

The study in [10] carried out the accelerated thermal aging experiment on slice samples of XLPE cable insulation at four different temperatures (165 °C, 145 °C, 125 °C and 115 °C), then measured and recorded the changing process of their retention rates of EAB. Meanwhile, previous literature has revealed that when the aging temperature of XLPE reaches or exceeds 160 °C, spherulites in XLPE become molten; the aging process of XLPE at this temperature is significantly different from that below 160 °C [18,19]. Thus, the aging results obtained at 145 °C, 125 °C and 115 °C were selected for analysis in this paper. Figure 1 presents the results of the retention rates of EAB versus time at the indicated temperatures for slice samples of XLPE cable insulation and the corresponding fitting curves.

The relationship between the degradation and temperature in Figure 1 was used to verify the applicability of the TTSP in XLPE materials. We firstly selected a reference temperature *T_ref_* = 115 °C, which is the lowest experimental temperature in the present case. Then, the results of the fitting curve at a second temperature *T* were used to calculate the corresponding multiplicative shift factor $\alpha_{T}$ according to the Equation (5), such that the multiplication of the times associated with the second temperature by $\alpha_{T}$ gives the best overlap (superposition) of the data from *T* to *T_ref_*. Taking the calculation procedure of data at 125 °C as an example, by referring to Equation (5), the ratio of $t_{ref}$ to *t_T_* was calculated when the retention rate of EAB was 80%, 70%, 60% and 50%; then, the average value was taken as the corresponding multiplicative shift factor $\alpha_{T}$, which was 1.63. This procedure was then completed on the data at 145 °C and the value of $\alpha_{T}$ = 5.15 was obtained. In addition, the multiplicative shift factor $\alpha_{T}$ = 1 was defined at 115 °C since the times at this temperature remained constant.

**Figure 2:**
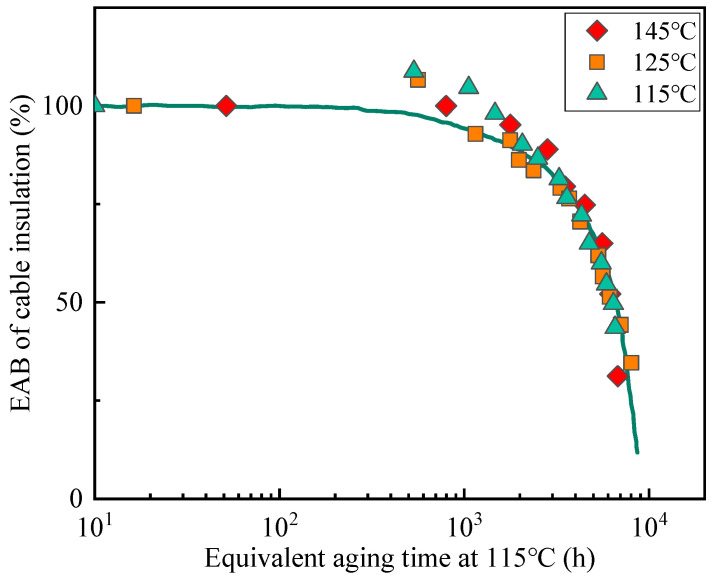
Time-temperature superposition of the elongation results from Figure 1 at 115 °C.

The resulting superposition and the empirical shift factors used to achieve superposition for the data from Figure 1 are shown in Figure 2. The time-temperature superposition approach was utilized for every experimental data point. It can be observed that the superposition is excellent as expected, given the similarities in curve shapes for the three aging temperatures in Figure 1, especially in the range where the retention rate of EAB is below 90%.

**Figure 3:**
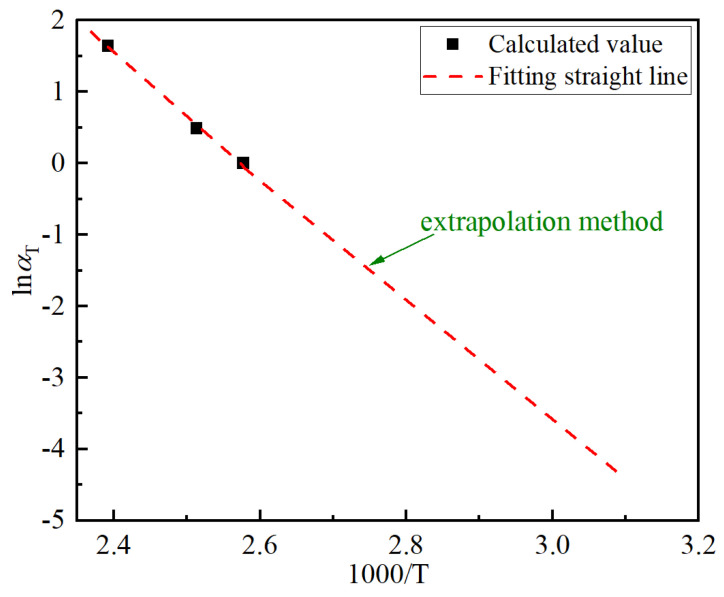
Arrhenius plot of the derived multiplicative shift factors for the EAB of slice samples of XLPE cable insulation.

The derived multiplicative shift factors could be plotted on an Arrhenius plot (the natural logarithm of $\alpha_{T}$ versus inverse absolute temperature) to see if Arrhenius behavior is indicated [13]. The Arrhenius plot was shown in Figure 3. By continuing the fitting to the data in Figure 3, the linearity is up to 0.997 and the linear fitting equation was presented in Equation (8).

(8) $$\log\alpha_{T}=23.14-8.99\times \frac{1000}{T}.$$

These results show that the log of the multiplicative shift factor $\alpha_{T}$ has an obvious linear relationship with the inverse absolute temperature T, which is basically consistent with the TTSP according to Equation (7). The observed line in Figure 3 could be extrapolated as shown, allowing predictions to be made at lower temperatures. Thus, the applicability of the Arrhenius equation in the transformation of the changing process of EAB of XLPE materials under different thermal aging conditions has been verified, and the TTSP can be the basis of a prediction methodology for the long-term behavior of XLPE materials based on short-term tests at different temperatures.

## 3. Experimental

### 3.1. Materials and Aging Experiments

Though scholars have observed the changing process of EAB of XLPE cable insulation, most of them are study the oven aging of slice samples. In fact, the aging process of slice samples might be not equivalent to that of the service-aged cables due to different oxygen conditions [14,15], which may not be able to be used as reference curves to predict the aging trends of the service-aged cables.

In this paper, three 8.7/10 kV single-core XLPE cables of a length of 4.5 m produced by Yuandong Company were selected as the research object in the aging experiments. The radius of the conductor was set to 10 mm and the insulation thickness was set to 4.5 mm. The silicone rubber heating strips tightly wrapped around the surface of the cable samples were chosen to heat the cable insulation evenly to accelerate the aging process. A total of 100 Pt thermal resistive sensors were placed between the heating strips and cable samples; they monitored the temperature information and transmitted the temperature signals to the PID temperature controller in real time. The PID temperature controller then sends the corresponding instructions to solid-state relays to realize the control of the cable temperature. The thermal aging treatment temperature of the cable insulation was 140 °C and the aging time lasted 80 days (d). Aging cable samples with a length of 1 m were cut from the aging system at the aging time points of 0 d, 10 d, 20 d, 35 d, 50 d, 65 d and 80 d. The cable insulation temperature during accelerated thermal aging experiments is considered to be homogeneous as it is heated externally.

**Figure 4:**
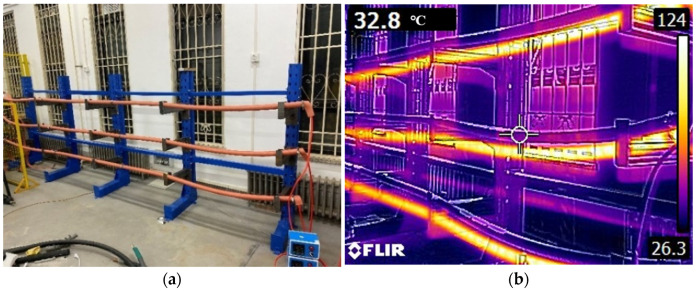
Physical picture of the heating platform and the infrared thermal image: (**a**) Physical picture of heating platform; (**b**) The infrared thermal image.

Figure 4 presents the physical picture of the heating platform and the infrared thermal image during the heating process. In order to ensure safety during the aging experiments, a custom-made metal holder was used to hold the cable samples.

### 3.2. Testing Method of EAB

**Figure 5:**
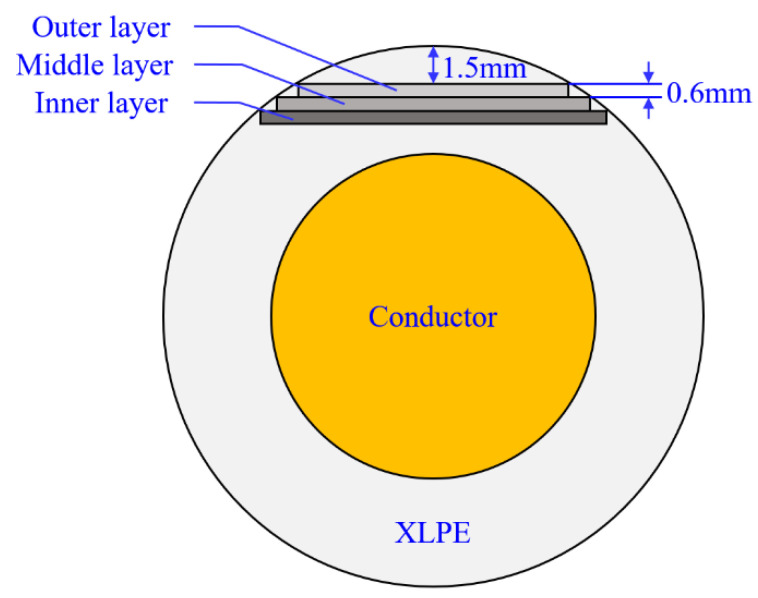
Schematic diagram of cable insulation crosscutting.

A crosscutting machine was used to cut outer, middle and inner insulation layer strips from aged cables in the experiments. Figure 5 presents the schematic diagram of cable insulation crosscutting.

The cutting process can be briefly described as follows: firstly, the XLPE layer strip with a thickness of 1.5 mm was cut off from the surface of the cable insulation; then three insulation layer strips with a thickness of 0.6 mm were cut from the remaining cable insulation one by one; finally, the layer strips were molded to dumbbell-shaped test specimens according to GB/T 2951.1-1997 and they were torn at a rate of strain of 50 mm/min by a tensile testing machine at 25 °C.

Considering the dispersion of EAB testing of specimens due to the sensitivity of EAB to the surface condition of samples, three dumbbell-shaped specimens were tested in each aged group and their mean value was recorded as the final value of EAB.

### 3.3. Results and Discussion

**Figure 6:**
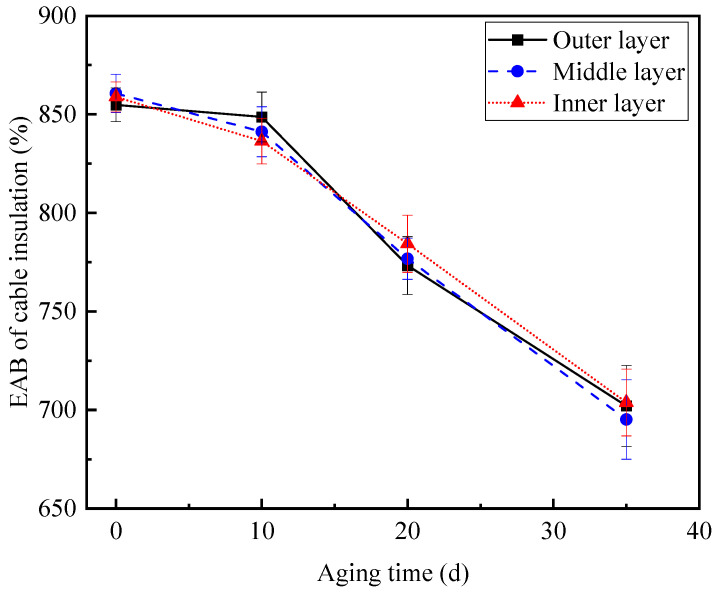
EAB and the corresponding variance of aging cable insulation in different positions versus the aging time.

Firstly, the mechanical properties of the dumbbell-shaped specimens of the outer, middle and inner insulation layers with an aging time of 0 d, 10 d, 20 d and 35 d were tested, respectively. The results of the EAB and the corresponding variance of the aging cable insulation at different positions versus aging time are presented in Figure 6.

The test results of the mechanical properties in Figure 6 illustrate that the overall change trend of the EAB of XLPE cable insulation has a good monotonic relationship with the aging time. The EAB of the aging cable insulation samples at different positions decreases with an increase in the number of aging days, which means that the aging phenomenon does occur in the cable insulation; the longer the aging time, the more significant the cable aging status. Meanwhile, it is notable that the EAB of the inner, middle and outer layers of the cable insulation are basically the same when their aging days are the same. In other words, the aging phenomenon occurs evenly in the cable insulation; the main reason for this is that the aging environments, such as the aging temperature and oxygen conditions, faced by the outer, middle and inner insulation layers are the same.

**Figure 7:**
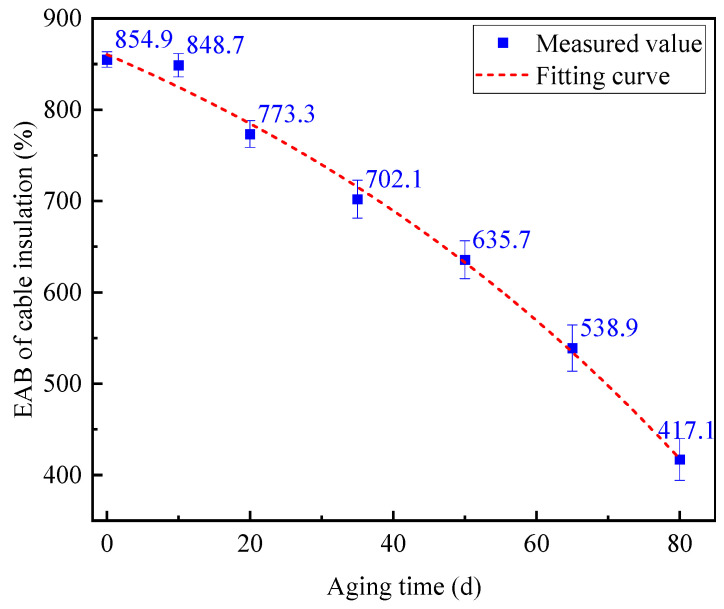
EAB and the corresponding variance of the aging middle cable insulation layer samples versus aging time.

Thus, when analyzing the mechanical properties of aged cables with a different aging time, it is not necessary to consider the influence of the cutting position of dumbbell-shaped specimens. Figure 7 shows the variance of the EAB of dumbbell-shaped specimens prepared from the middle insulation layers with an aging time from 0 d to 80 d. It can be seen that the EAB of the aging cable insulation still decreases with the extension of aging time, and even decreases to 417.1% when the aging time is 80 d. In fact, a failure criterion for the mechanical property of the cable insulation has been proposed for a few years; that is, the XLPE cable is considered to reach the end of its life when the EAB of the insulation reaches 50% absolute elongation [8]. According to the failure criterion, one conclusion could be reasonably drawn that the cable with the aging time of 80 d has exceeded its service life since its absolute elongation has been reduced to 48.5% of that without aging.

Some scholars have studied the relationship between the mechanical properties of cable insulation and the aging time, and the exponential function relationship between EAB and the aging time *t* through curve fitting was deduced, which can be fitted by Equation (9) [14,20]:

(9) $$\mathrm{EAB}=a\times e^{bt}+c,$$

where *a*, *b* and *c* are undetermined constants. Then, according to formula (9), the data of the EAB with aging time t in Figure 7 were further fitted by a software, and the final fitted curve is shown in Figure 7, represented by the red dashed line. The fitting function expression obtained is presented in Equation (10), and the corresponding fitting degree had a value of *R*^2^ = 0.994. The above results indicate that the fitting result could describe the thermal aging process of XLPE cable insulation well, which could be used as a reference curve to predict the thermal aging trend and residual life of XLPE cables in operation.

(10) $$\mathrm{EAB}=-408.35\times e^{0.00919t}+1272.87$$

### 3.4. Calculation Method for the Multiplicative Shift Factor Based on the TTSP

The traditional prediction process of the aging trend and residual life of cables based on the TTSP can be briefly summarized as follows:

(1) Accelerated aging tests on cables are carried out at a certain temperature (set as the reference temperature), and the relationship curve of the characteristic property (such as the EAB) versus aging time is established through measurements, which is set as the baseline prediction curve of the aging trend.
(2) The accelerated thermal aging experiment on cables are further carried out at several other temperatures, and the time at which the cable performance deteriorates to the end of its life (lifetime) at different temperatures is further measured and calculated. By calculating the ratios of the lifetime at the reference temperature to the lifetime at each aging temperature, the corresponding multiplicative shift factor $\alpha_{T}$ is obtained. Then, the relationship of the natural logarithm of the multiplicative shift factor versus the inverse absolute temperature *T* at the above temperature conditions can be drawn under a coordinate system. Since the Arrhenius extrapolation assumption is valid, the prediction of the multiplicative shift factor $\alpha_{T}$ at a certain temperature can be deduced by linear fitting.
(3) By combining the multiplicative shift factor corresponding to a certain temperature and baseline prediction curve of the aging trend, the predicted behavior of the characteristic property versus the aging time would be available by simply multiplying the times by $\alpha_{T}$, based on which the prediction of the aging trend and residual life of the cables would be achieved.

However, the temperature distribution in the insulation layer of XLPE distribution cables laid underground along lines is uneasy to acquire. As a result, it is difficult to accurately obtain the corresponding multiplicative shift factor $\alpha_{T}$ by Arrhenius equation extrapolation with the absence of the accurate temperature, hindering the direct conversion of aging trends by the use of temperature information and the engineering application in this method.

**Figure 8:**
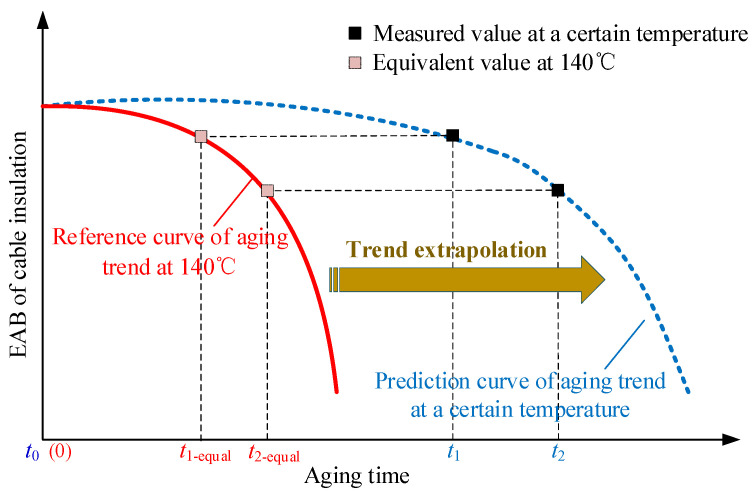
Schematic diagram of the calculation for the multiplicative shift factor at a certain temperature.

To solve this problem, a calculation method for the multiplicative shift factor of XLPE distribution cables was proposed. According to the basic idea of the TTSP, for XLPE cable insulation, in which the Arrhenius extrapolation assumptions have been verified to be applicable, the degradation curves at different aging temperatures would have the same shape when plotted versus the log of the aging time, and could be converted to each other by using empirical shift factors. That is, the ratio of the time spent when the EAB of the cable decreases to an arbitrary value at a certain aging temperature to the time spent when the EAB of the cable decreases to the same value at the reference temperature is equal to the corresponding multiplicative shift factor $\alpha_{T}$. Thus, taking the schematic diagram in Figure 8 for example, the following calculation process for $\alpha_{T}$ can be conducted. Firstly, the specific time *t*_0_ when the cable is put into operation can be obtained by querying the cable installation files. Then the value of the EAB of the cable insulation (EAB_1_) can be measured and obtained by taking samples from service-aged cables during the period of maintenance, and the corresponding time is recorded as *t*_1_. After that, The EAB_1_ value is substituted into the reference trend curve of cable thermal aging to calculate the corresponding aging time spent *t*_1-equal_. Finally, the multiplicative shift factor $\alpha_{T}$ can be obtained by the ratio of *t*_1-equal_ to (*t*_1_ − *t*_0_).

Moreover, even if the cable installation files are unavailable, the above idea could still be used to obtain the multiplicative shift factor $\alpha_{T}$. For example, as shown in Figure 8, another value of the EAB of the cable insulation (EAB_2_) can be obtained by taking samples from service-aged cables during the period of maintenance, and the corresponding time is recorded as *t*_2_. Similarly, the EAB_2_ value is substituted into the reference trend curve of cable thermal aging to calculate the corresponding aging time spent *t*_2-equal._ Then the multiplicative shift factor $\alpha_{T}$ can also be obtained by the ratio of (*t*_2-equal_ − *t*_1-equal_) to (*t*_2_ − *t*_1_).

If the multiplicative shift factor $\alpha_{T}$ is equal to *y* at a certain temperature *T*, according to the Equation (10), the corresponding thermal aging trend of XLPE cables can be deduced as shown in Equation (11)

(11) $$t^{\prime }=\frac{\ln(\frac{x-1272.87}{-408.35})}{0.00919}÷y,$$

where *x* represents the EAB of the measured cable insulation and *t*′ represents the corresponding aging time at temperature of *T*.

By referring to the proposed failure criterion for the mechanical property of the cable insulation [8], in which the XLPE cable is considered to reach the end of its life when the EAB of the insulation reaches 50% absolute elongation, the whole life time (*t*″) of service-aged XLPE cables at a temperature *T* can be calculated further, which is presented in Equation (12)

(12) $$t^{\prime \prime }=\frac{\ln(\frac{430.35-1272.87}{-408.35})}{0.00919}÷y=\frac{78.81}{y},$$

where *t*″ represents the whole life time of service-aged XLPE cables at temperature *T*. Finally, the residual life of XLPE cables $t_{rest}$ can be calculated as follows:

(13) $$t_{rest}=t^{\prime \prime }-t^{\prime }=\frac{78.81}{y}-\frac{\ln(\frac{x-1272.87}{-408.35})}{0.00919y}.$$

**Figure 9:**
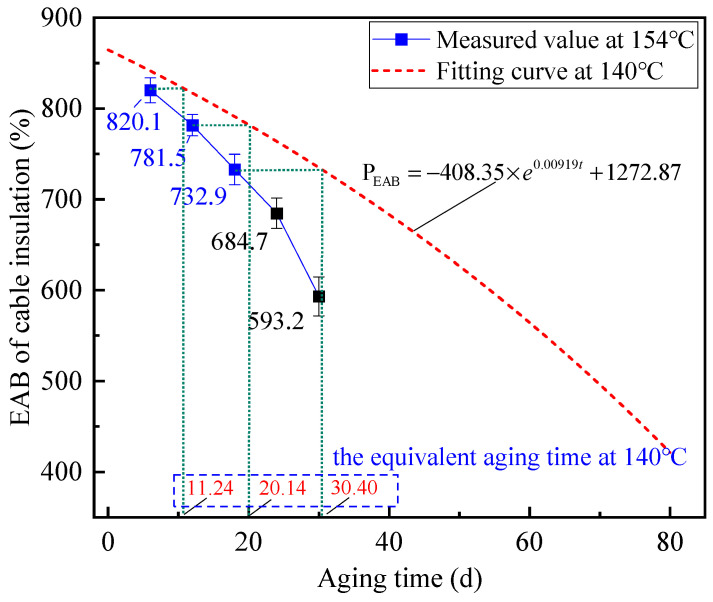
The EAB and the equivalent shift process of the aging cable insulation versus aging time at 154 °C.

In order to verify and analyze the effectiveness of the above proposed calculation method to find the multiplicative shift factor, firstly, the accelerated thermal aging experiments of XLPE cables at 154 °C were carried out based on the heating platform shown in Figure 4. The aging time of cable samples was set to 6 d, 12 d and 18 d, respectively, and the EAB of the cable insulation after aging was tested. The measured results of the EAB are shown in Figure 9, represented by the blue dots.

It can be clearly observed that the EAB of the cable insulation decreases with the extension of the aging time, which is 820.1%, 781.5% and 732.9% when the aging time is 6 d, 12 d and 18 d, respectively. Meanwhile, the fitting curve of the variance of the EAB at 140 °C is also drawn in Figure 9, as represented by the red dashed line. It is worth noting that the slope of the EAB at the aging temperature of 154 °C is bigger than that at 140 °C, indicating that the aging speed of XLPE cable insulation is improved by the increase of aging temperature.

According to the prediction method of the aging trend of the XLPE cable insulation proposed previously, the multiplicative shift factor with aging time of 6 d, 12 d and 18 d can be calculated respectively using the ratio of the corresponding equivalent aging time at 140 °C to the actual aging time at 154 °C. Some data involved in the calculation process and the corresponding multiplicative shift factors calculated are presented in Table 1.

The multiplicative shift factors deduced based on the TTSP are 1.873, 1.678 and 1.689 when the aging time is 6 d, 12 d and 18 d, respectively. The standard deviation of these three data points is 0.089, demonstrating the stability of the proposed method for converting the aging trends at various temperatures. Then, the average value was further calculated and set as the final multiplicative shift factor at the aging temperature of 154 °C, which was 1.747. On this basis, combined with the prediction equation for the aging trend at 140 °C in Equation (10), the EAB of aged cable insulation with the aging time of 24 d and 30 d at 154 °C can be respectively deduced and predicted, as shown in Table 2.

From Table 2, it can be seen that the EAB prediction values of the thermal-aged cable insulation with the aging time of 24 d and 30 d at 154 °C are 672.6 and 611.9, respectively. Meanwhile, to verify the accuracy of the prediction results for the XLPE cable’s mechanical property, the accelerated thermal aging experiments of XLPE cables with the aging time of 24 d and 30 d at 154 °C were further carried out. The experimental results show that the actual EAB of thermal-aged XLPE cables with the aging times of 24 d and 30 d at 154 °C is 684.7 and 593.2, respectively, as represented by black dots in Figure 9. By comparing the predicted and measured values of the EAB, it reveals that the prediction method proposed by this paper is able to predict the changing trend of the EAB on XLPE cable insulation under thermal aging fairly well, since the deviations of the prediction results are 1.77% and 3.15% when the aging time is 24 d and 30 d, respectively.

At the same time, according to Equation (13) and the measured value of the EAB of thermal-aged XLPE cables with the aging time of 18 d, the prediction value of the residual life of the XLPE cable insulation with the thermal aging time of 18 d at 154 °C was calculated, as shown in Table 2.

Combined with the predicted values of the EAB of thermal-aged XLPE cables with the aging time of 24 d and 30 d at 154 °C, as well as the final multiplicative shift factor calculated above, the predicted value of the residual lives of thermal-aged XLPE cable with the aging time of 24 d and 30 d at 154 °C can be predicted by reference to Equation (11). The prediction results are also recorded in Table 2, where the corresponding residual life prediction values of the aged cable insulation are 21.15 d and 15.12 d, respectively. According to Table 2, it can be noted that the reduction in predicted residual life of thermal-aged XLPE cables at 154 °C when the aging time has a value of 18 d to 24 d is 6.59 d, while the reduction in the true residual life is 6 d, indicating that the prediction error is 9.8%. Similarly, when the aging time has a value of 18 d to 30 d, the reduction in the predicted residual life of thermal-aged XLPE cables at 154 °C is 12.62 d, indicating that the prediction error is 5.2%. Therefore, the prediction error for residual life is within 10%. Through the experimental verification above, one conclusion that can be confidently drawn is that the prediction method proposed for the thermal aging trend and residual life of XLPE cables based on the EAB is efficient.

## 4. Realization Process of Non-Destructive Residual Life Prediction

### 4.1. Input Impedance of Power Cables

**Figure 10:**
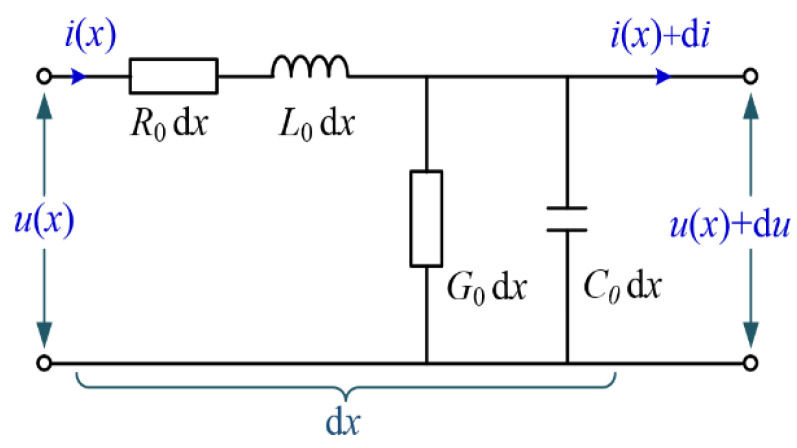
Distributed parameter model of the power cable.

As illustrated in Figure 10, the distribution parameter model could be used to describe the electrical network structure of power cables under a high frequency power source according to the transmission line theory [21]. *R*_0_, *L*_0_, *G*_0_ and *C*_0_ represent the equivalent resistance, inductance, conductance and capacitance of the power cable per unit length, respectively, and they can be calculated according to the material, size, structure and other parameters of the power cables. *x* is the distance from the cable head end.

For one even transmission line with a length of *l* and an open end, the input impedance at the head end *Z_in_* can be presented as

(14) $$Z_{in}=Z_{0}\left(\frac{1+e^{-2\gamma l}}{1-e^{-2\gamma l}}\right),$$

where *Z*_0_ and *γ* are the characteristic impedance and propagation constant, respectively, which are given by:

(15) $$Z_{0}=\sqrt{\left(R_{0}+j\omega L_{0})/(G_{0}+j\omega C_{0}\right)};$$

(16) $$\gamma =\alpha +j\beta =\sqrt{\left(R_{0}+j\omega L_{0}\right)\left(G_{0}+j\omega C_{0}\right)}.$$

*α* and *β* are the attenuation coefficient and propagation coefficient, respectively, *ω* is the angular frequency, which can be obtained using Equation (17)

(17) ω=2πf,

where *f* is the signal frequency.

It is notable that the literatures [22,23] reveal that the values of the characteristic impedance *Z*_0_ and propagation constant *γ* of aged cables are decided by *G*_0_ and *C*_0_. Since *R*_0_ and *L*_0_ are almost unchanged if a degradation occurs, *G*_0_ + *jwC*_0_ can be derived from the complex dielectric constant *ε*(*ω*). Meanwhile, the complex dielectric constant of XLPE material in the frequency range between 10 kHz and 100 MHz can be fitted according to the Cole–Cole model [21,24], as shown in Equation (18):

(18) $$\varepsilon \left(\omega \right)=\frac{C\varepsilon_{0}}{1+D{\left(i\omega \right)}^{p}},$$

where, *C*, *D* and *P* are the fitting coefficients, *P* ∈ (0, 1) and *ε*_0_ is the vacuum dielectric constant.

### 4.2. Identifing the Number and Distribution of Local Aging Segments

BIS measuring has been developed in recent years, and has become an effective method of recognizing and diagnosing the local degradation in insulation cables [21,22]. The principle of BIS measuring is to measure the input impedance of the cable in a wide frequency range, which is closely related to the distribution of the propagation coefficient and the characteristic impedance of the cable; in other words, BIS measuring contains healthy information about the cable insulation state. Since impedance spectroscopy could be easily measured by an impedance analyzer at a low voltage, such as 1 V, it means that BIS measurement is non-destructive.

It has been proved that the input impedance spectrum of a coaxial cable varies approximately periodically with the signal frequency *f* [21,22]. At high frequencies (*ωC*_0_ >> *G*_0_, *ωL*_0_ >> *R*_0_), *Z*_0_ is almost a constant; according to Equation (14), it could be reasonably deduced that the periodicity of the input impedance spectrum is determined by e^−2*γl*^. If another defect was located at position *x* = *l*_1_, then the change of the corresponding distribution parameter would occur, and the periodicity of the input impedance spectrum would also be influenced by e^−2*γl*_1_^ [25]. Referring to the Euler equation, the parameter e^−2*γl*^ can be expanded as follows:

(19) $$e^{-2\gamma l}=e^{-2\alpha l}\times \left[\cos\left(\frac{4\pi f}{v}l\right)-j\sin\left(\frac{4\pi f}{v}l\right)\right].$$

Since *α* is the increasing function of *f*, *e*^−2*γl*^ can be regarded as an approximate periodic signal with $f_{x=l}^{\prime }$ = 2*l*/*v* as the equivalent frequency, although the amplitude is a decreasing function of the signal frequency *f*. Similarly, *e*^−2*γl*_1_^ can be regarded as an approximate periodic signal with $f_{x=l_{1}}^{\prime }$ = 2*l*_1_/*v* as the equivalent frequency. Then, the ratio of $f_{x=l_{1}}^{\prime }$ to $f_{x=l}^{\prime }$ can be given as follows:

(20) $$f_{x=l_{1}}^{\prime }/f_{x=l}^{\prime }=(2l_{1}/v)/(2l/v)=l_{1}/l.$$

Then, *l*_1_ can be calculated by Equation (21):

(21) $$l_{1}=l\times \frac{f_{x=l_{1}}^{\prime }}{f_{x=l}^{\prime }}$$

For any local aging cable segment, the two ends of which belong to impedance discontinuities. Thus, the characteristic parameters, such as the amplitude or phase spectrum, of the input impedance can be firstly measured, and then the equivalent frequency *f*′ corresponding to the two ends of the aging cable segments and the end of the cable could be obtained by using Integral transformation or Fourier transform [3,22]. On this basis, the number and distribution of local aging cable segments can be identified according to Equation (21).

### 4.3. Velocity Calculation of Local Aging Cable Segments Based on BIS Measuring

**Figure 11:**
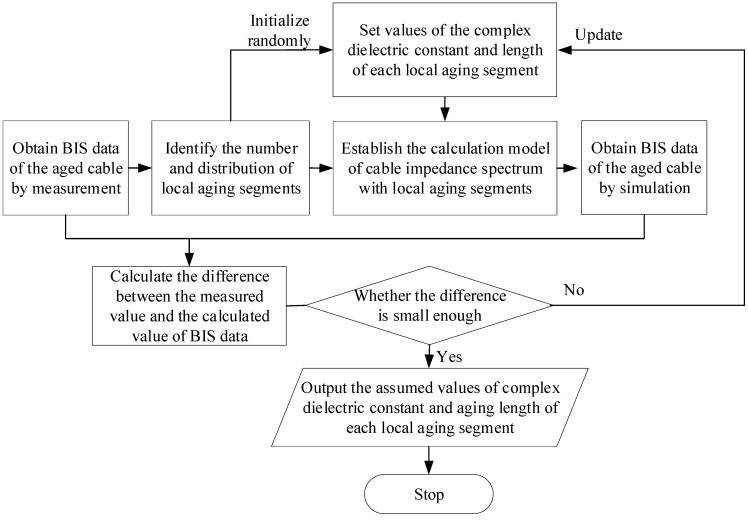
Acquisition procedure of the complex dielectric constant for each local aging segment based on BIS measuring combined with PSO [22].

Zhou Zhiqiang et al. [22] have succeeded in the acquisition of the complex dielectric constant for each local aging cable segment by BIS measuring combined with particle swarm optimization (PSO). The corresponding operation flow diagram of is shown in Figure 11.

Firstly, BIS data of the aged cable could be obtained by measurement, then the number and distribution of local aging cable segments would be identified according to the literature [21]. Then values of the complex dielectric constant and length of each local aging segment are randomly set (each cable segment can be described by four unknown parameters, including constants *C*, *D*, *P* and length *l*). It should be noted that the calculation model of the cable impedance spectrum with local aging segments would be established and then the calculated values of BIS data would be obtained. After that, the difference between the measured value and the calculated value of BIS data are calculated; if it is not small enough (usually being compared with a pre-set termination criterion), values of the complex dielectric constant and the length of each local aging segment would be reset, and the previous steps would be repeated. The iteration is carried out until the termination criterion is met. In this situation, the values of the complex dielectric constant and aging length of each local aging segment would be output, which is regarded as the corresponding actual values.

Meanwhile, other literature [26] has revealed that the wave velocity in a XLPE cable could reflect its aging status. Generally, the more serious the aging status of the cable, the smaller the value of the wave velocity. The propagation speed *v* of high frequency electromagnetic waves in a XLPE cable is independent of frequency, which is calculated as follows:

(22) $$v\approx \frac{c_{0}}{\sqrt{\mu_{r}\varepsilon_{r}}},$$

where *ε_r_* is the real part of the complex dielectric constant of cable insulation, *μ_r_* is the relative permeability of the cable conductor and *c*_0_ is the speed of light. According to the above research results, the wave velocity in each local aging cable segment can be obtained.

### 4.4. Non-Destructive Residual Life Prediction

**Figure 12:**
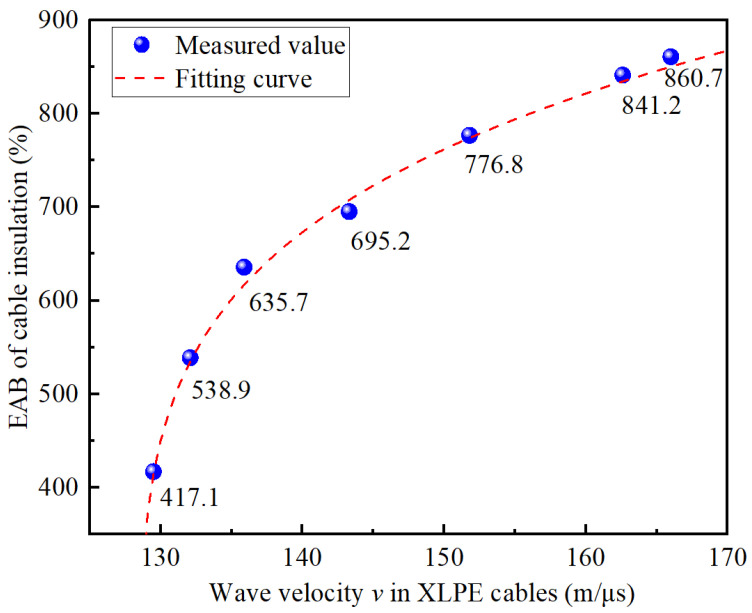
Relation curve of the EAB of the cable insulation versus wave velocity in XLPE cables.

It has been found that there is a monotonic relationship between the wave velocity in the cable and the corresponding EAB value [26]. Referring to the accelerated aging experiment condition and testing process in literature [26], the relationship between the wave velocity in the cable and the corresponding EAB value was further established, as shown in Figure 12.

By referring to the form of the fitting Equation (9) and considering the changing trend of the cable wave velocity with aging time, the data of the EAB versus the wave velocity *v* in Figure 12 were further fitted by software, and the final fitted curve is shown in Figure 12, represented by the red dashed line. The fitting function expression obtained is presented in Equation (23), and the corresponding fitting degree value was *R*^2^ = 0.9966. The above results indicate that the fitting result could describe the relationship well, which could be used to convert the wave velocity *v* calculated to the corresponding EAB value.

(23) $$\mathrm{EAB}=420.1\times e^{0.1962\times \ln\left(v-128.5\right)}-5.2.$$

Thus, the EAB along the cable line could be obtained by using the value of wave velocities in the local cable aging segments obtained according to Equation (23). On this basis, combined with the research results achieved in Section 3, the non-destructive residual life prediction of each aging cable segment in the non-uniform aging XLPE distribution cables can be realized.

## 5. Discussion

In fact, the temperature of the XLPE distribution cable in actual operation is not constant due to the effect of factors such as the temperature difference between day and night, seasonal climate and line load. Since the range of the temperature change is not too large, and considering the fact that the cable operating life is usually long and mainly counted in years, the operating temperature of the cable body is assumed to be a certain value (i.e., a long-term equivalent aging temperature) in the process of predicting the aging trend of XLPE cables [25]. This above treatment helps to simplify the aging trend prediction model and enhance its practicability.

In addition, it needs to be noted that the proposal of non-destructive prediction methods for the thermal aging trend and residual life of XLPE cables is the ultimate researching objective for the operation and maintenance management of XLPE distribution cables. The main purpose of this paper is to propose a method, which could realize the accurate prediction of the thermal aging trends and residual life of XLPE cables. Though the prediction method proposed in paper is still destructive to some extent, it could be further developed easily into a non-destructive method in combination with the existing research results. The literature [21] has revealed that the wave velocity in the XLPE cable could reflect its aging status, which could be obtained through non-destructive measurement. Generally, the more serious the aging status of the cable, the smaller the value of wave velocity. Meanwhile, it has been found that a monotonic relationship between the wave velocity in the cable and the corresponding EAB value can be established. In view of this, experiments could be carried out to obtain the above relationship; then the non-destructive prediction could be realized, based on the measurement of the wave velocity.

## 6. Conclusions

Aging performance degradation modeling and non-destructive life prediction methods of XLPE distribution cables are the focus of research in distribution network operation and maintenance. This paper proposed a residual life prediction method for XLPE distribution cables based on the TTSP by non-destructive BIS measuring on site. The main conclusions are as follows:

(1) The applicability of the TTSP in the transformation of the changing process of EAB of XLPE at different thermal aging temperatures was verified based on the Arrhenius equation. Thus, the TTSP can be the basis of a prediction methodology for the long-term behavior of XLPE materials based on short-term tests at different temperatures.
(2) The relationship between the EAB of XLPE cables and the aging time at 140 °C was established and well fitted by an equation, which could be used as a reference curve to predict the thermal aging trend and residual life of service-aged XLPE cables.
(3) A calculation method for predicting the aging trend of service-aged cables using the changing process of EAB was proposed, in which the corresponding multiplicative shift factor can be obtained based on the TTSP instead of Arrhenius equation extrapolation. Moreover, the availability of the prediction method was further proved through experiment. The prediction error for the cable’s EAB value was no more than 3.15% and the prediction error for residual life was within 10% in this case.
(4) The realization process of non-destructive residual life prediction combined with BIS measuring on site was described in detail, and the relationship between the wave velocity in the cable and the corresponding EAB value was established.

## Figures and Tables

**Table 1:** Multiplicative shift factor for results of the EAB of the thermal-aged cable insulation at 154 °C.

Actual Aging Time (d)	EAB (%)	Equivalent Aging Time (d)	Multiplicative Shift Factors Calculated
6	820.1	11.24	1.873
12	781.5	20.14	1.678
18	732.9	30.40	1.689

**Table 2:** Prediction values of the EAB and the residual life of the thermal-aged cable insulation at 154 °C.

Actual Aging Time (d)	EAB Prediction Values of the Aged Cable Insulation (%)	Residual Life Prediction Values of the Aged Cable Insulation (d)
18	-	27.76
24	672.6	21.15
30	611.9	15.12

## Data Availability

Not applicable.
